# Wigner-molecularization-enabled dynamic nuclear polarization

**DOI:** 10.1038/s41467-023-38649-5

**Published:** 2023-05-23

**Authors:** Wonjin Jang, Jehyun Kim, Jaemin Park, Gyeonghun Kim, Min-Kyun Cho, Hyeongyu Jang, Sangwoo Sim, Byoungwoo Kang, Hwanchul Jung, Vladimir Umansky, Dohun Kim

**Affiliations:** 1 Department of Physics and Astronomy, and Institute of Applied Physics, Seoul National University, Seoul, 08826 Korea; 2 Department of Physics, Pusan National University, Busan, 46241 Korea; 3 Braun Center for Submicron Research, Department of Condensed Matter Physics, Weizmann Institute of Science, Rehovot, 76100 Israel

**Keywords:** Qubits, Quantum dots

## Abstract

Multielectron semiconductor quantum dots (QDs) provide a novel platform to study the Coulomb interaction-driven, spatially localized electron states of Wigner molecules (WMs). Although Wigner-molecularization has been confirmed by real-space imaging and coherent spectroscopy, the open system dynamics of the strongly correlated states with the environment are not yet well understood. Here, we demonstrate efficient control of spin transfer between an artificial three-electron WM and the nuclear environment in a GaAs double QD. A Landau–Zener sweep-based polarization sequence and low-lying anticrossings of spin multiplet states enabled by Wigner-molecularization are utilized. Combined with coherent control of spin states, we achieve control of magnitude, polarity, and site dependence of the nuclear field. We demonstrate that the same level of control cannot be achieved in the non-interacting regime. Thus, we confirm the spin structure of a WM, paving the way for active control of correlated electron states for application in mesoscopic environment engineering.

## Abstract (web summary)

Wigner molecules, or correlated localized electron states, has been reported in semiconductor quantum dots, but their interaction with environment has been less explored. Here the authors use the spin multiplet structure of a three-electron Wigner molecule to enhance and control dynamic nuclear polarization.

## Introduction

Semiconductor quantum dot (QD) systems facilitate investigations of the interaction between electron spins and nuclear environments, which is known as the central-spin problem^1,2^. Although the fluctuation of nuclear fields, which is quantified by the effective Overhauser field *B*_nuc_^3,4^, often acts as a magnetic-noise source for spin qubits^3^, the hyperfine electron–nuclear spin interaction allows achieving dynamic nuclear polarization (DNP)^5–8^. DNP is used for enhancing the signal-to-noise ratio in nuclear magnetic resonance^6^ and prolonging coherence times in QD-based spin qubits^9,10^. Gate-defined semiconductor QDs have been used to achieve the fast probing of nuclear environments^8,11,12^, bidirectional DNP^11^, and active feedback control of nuclear fields^10^.

While the DNP achieved by spin-flip mediated transport with an applied bias^13,14^ allows large DNP^13^, the QD - reservoir tunnel rate needs to be large enough to allow the finite spin-flip current. On the contrary, the DNP based on the pulsed-gate technique can be demonstrated while maintaining the small tunnel rates ~10^1^ kHz. Because the qubit control typically requires small QD-reservoir tunnel rates transition from the pulsed-gate DNP to qubit experiments is straightforward without additional parameter modulation via the gate voltages. However, spin qubit control combined with DNP has been limited to two-electron singlet–triplet (ST_0_) spin qubits^9–12,15^. Despite the versatility of gate-defined QD systems^16–19^, the large singlet–triplet energy splitting *E*_ST_ (~10^2^ *h*·GHz; *h* is Planck’s constant) in particular in GaAs limits the access to higher spin states^20^ in multielectron QDs at moderate external magnetic fields *B*_0_ < 1 T or within a typical frequency bandwidth of experimental setups.

Coulomb-correlation-driven Wigner molecules (WMs) in confined systems^21–25^ may provide new directions for expanding nuclear control to multielectron systems. Recent studies on QDs in various systems have shown clear evidence of WM formation^22,23,25–29^. It has been demonstrated that the *E*_ST_ can reach down to ~10^0^ *h*·GHz upon the WM formation^27,29^ because of strong electron–electron interactions confirmed by full-configuration interaction (FCI)-based theories^23,25,28,30^. However, most studies have focused on the spectroscopic confirmation of WM formation, and studies on the open system dynamics using correlated states have not been reported to date.

Here, we demonstrate the formation of a WM in semiconductor QDs, which helps achieving efficient spin environment control. We use gate-defined QDs in GaAs and exploit the quenched energy spectrum of the WM (*E*_ST_ ~ 0.9 *h*·GHz) to enable mixing between different spin subspaces within *B*_0_ < 0.3 T. Furthermore, we demonstrate DNP by pulsed-gate control of the electron spin states. Leakage spectroscopy and Landau–Zener–Stuckelberg (LZS) oscillations confirm a sizable bidirectional change in *B*_nuc_ ~ 80 mT and the spatial Overhauser field gradient Δ*B*_nuc_ ~ 35 mT due to the long nuclear spin diffusion time *τ*_N_ ~ 62 s. Further, we demonstrate on-demand control of *B*_nuc_ combined with coherent LZS oscillations, providing a new route for realizing controllable DNP using correlated electron states.

## Results

**Fig. 1:**
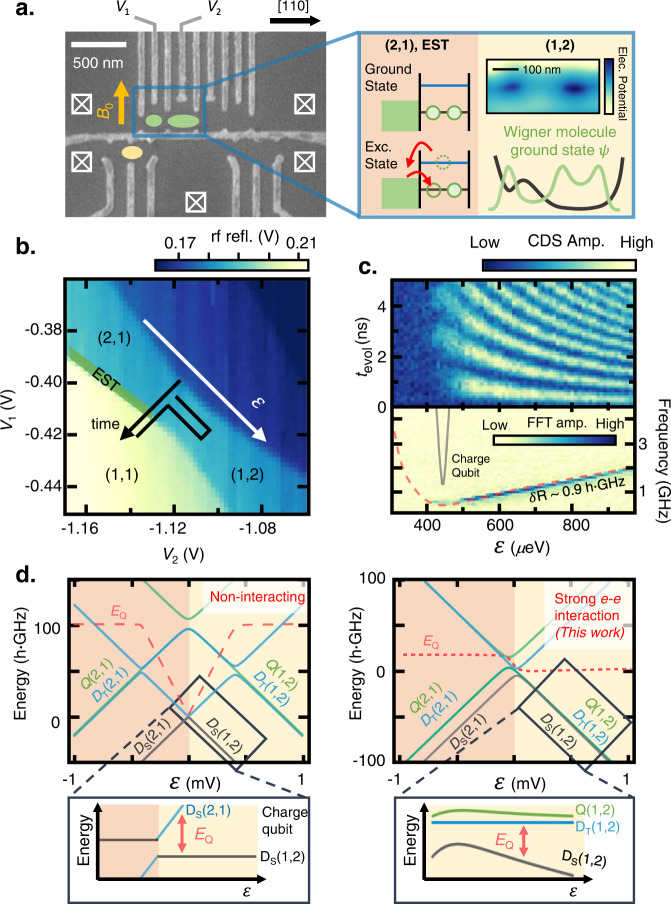
Wigner molecule formation in a GaAs double quantum dot. **a** Scanning electron microscope image of a GaAs quantum dot (QD) device similar to the one used in the experiment. Green dots denote the double QD defined for Wigner molecule (WM) formation which is aligned along the [110] crystal axis (black arrow). The inner plunger gate *V*_2_ is designed to have anisotropic confinement potential as shown in the right panel to facilitate the localization of the electronic ground state. Yellow circle: a radio-frequency (rf) single-electron transistor (rf-SET) charge sensor for rf-reflectometry. External magnetic field *B*_0_ is applied along the direction denoted by the yellow arrow. **b** Charge stability diagram of the double QD near the three-electron region spanned by *V*_1_ and *V*_2_ gate voltages. Green-shaded region: the energy-selective tunneling (EST) position for the state readout and initialization. **c** Landau–Zener–Stückelberg (LZS) oscillation of the WM at *B*_0_ = 0 T. The relative phase evolution between the excited doublet (*D*_T_) and the ground doublet (*D*_S_) results in the oscillation captured by the EST readout. Red-dashed curve in the fast Fourier transformed (FFT) map shows energy dispersion calculated from the toy-model Hamiltonian. The calculation yields quenched orbital energy spacing of the inner dot *δR* ~ 0.9 *h*·GHz. **d** Left (Right) panel: Energy spectrum along the (2,1)–(1,2) charge configuration in the non-interacting (strongly interacting, this work) regime with *δL* ~ 100 *h*·GHz (*δL* ~ 19 *h*·GHz), and *δR* ~ 100 *h*·GHz (*δR* ~ 0.9 *h*·GHz). *E*_Q_ (red-dashed curve) is the energy splitting between the two lowest levels.

Figure 1a shows a gate-defined QD device fabricated on a GaAs/AlGaAs heterostructure, where a 2D electron gas (2DEG) is formed ~70 nm below the surface (see Methods). We focus on the left double QD (DQD) containing three electrons. We designed the *V*_2_ gate to form an anisotropic potential, which is predicted to promote WM formation^22^. An electrostatic simulation of the electric potential at the QD site near *V*_2_ shows an oval-shaped confinement potential with anisotropy exceeding 3 (Fig. 1a, right panel). This potential can be tuned by the gate voltage, allowing the controlled electron correlation and localization of the ground state wavefunction within the DQD^22,24,26,27^. The yellow dot in Fig. 1a. denotes a radio-frequency single-electron transistor (rf-SET) charge sensor utilized for quantum state readout^31–33^. The device was operated in a dilution refrigerator with a base temperature of ~40 mK, an electron temperature *T*_e_ ~ 150 mK (Supplementary Note 1), and a variable *B*_0_ applied to the direction shown in Fig. 1a.

The three-electron DQD results in two spin doublets and one spin quadruplet state. Without the magnetic field in the (2,1) [(1,2)] charge configuration, doublet-singlet state *D*_S_(2,1) [*D*_S_(1,2)] with total spin *S* = 1/2 form the ground state where the two electrons in the left [right] QD form a spin singlet state and fill the ground orbital in the left [right] QD. Here, n [m] denotes the number of electrons in the left [right] QD by (n, m). When the two electrons in the left [right] QD form a spin triplet state and fill the excited orbital in the left [right] QD, the three-electron state result in either the doublet-triplet state *D*_T_(2,1) [*D*_T_(1,2)] with *S* = 1/2 or the quadruplet state *Q*(2,1) [*Q*(1,2)] with *S* = 3/2. Because of the orbital splitting, the *D*_T_, and *Q* states usually have higher energy compared to *D*_S_ states^34–36^. If a finite magnetic field is applied, doublet states with *S* = 1/2 are split into *m*_s_ = +1/2 and −1/2 states whereas quadruplet states with *S* = 3/2 are split into *m*_s_ = +3/2, +1/2, −1/2, and −3/2 states. Here *m*_s_ is the spin quantum number related to the *z* component of the electron spin angular momentum. The explicit spin structures are shown in the Methods. Hereafter, (n, m; *m*_s_) notation is used to describe both the charge configuration and spin angular momentum of a state.

First, we show the spectroscopic evidence of the WM at *B*_0_ = 0 T by probing *E*_ST_ in the right QD *δR*. Figure 1b shows a charge stability diagram around (2,1) and (1,2). The green-shaded region near the (2,1)–(1,1) charge transition is exploited for energy-selective tunneling (EST) readout and state initialization^27,37,38^. We tune the electron tunneling-in (-out) time *τ*_in_ (*τ*_out_) of the left dot to 14 (7) *μ*s. Starting from the initialized ground doublet state *D*_S_ in the (2,1) charge configuration, we apply non-adiabatic pulses (Fig. 1b) simultaneously to *V*_1_ and *V*_2_ with a rise time of ~500 ps and a repetition period of 51 μs ≫ *τ*_in_ to induce coherent LZS oscillation^39,40^. The oscillation reveals the relative phase evolution between the excited and ground doublet states (*D*_T_ and *D*_S_), the frequency of which is governed by *δR*.

Figure 1c shows the resultant LZS oscillations as a function of evolution time *t*_evol_ and detuning *ε*. The *E*_ST_ in GaAs DQDs in the non-interacting regime is typically on the order of 10^2^ *h.*GHz^20^ (Fig. 1d). In a charge qubit regime, a steep rise in the LZS oscillation frequency *f*_LZS_ ~ *E*_Q_/*h* as a function of *ε* (Fig. 1c, black curve) and short coherence time *T*_2_* ~ 10 ps due to strong susceptibility to charge noise is expected^41^. *E*_Q_ is the energy splitting between the two lowest levels (Fig. 1d, red-dashed curve). However, we find a significantly smaller *f*_LZS_ in the (1,2) charge configuration and *T*_2_* ~ 10 ns because of the reduced dispersion of *f*_LZS_ versus *ε*. This is reminiscent of a QD hybrid qubit^27,40,42^, but the excited energy is suppressed owing to the electron–electron interaction. WM formation in our previous GaAs device has been recently confirmed by FCI calculation^27,28,30^. Although such calculation is needed to rigorously determine parameters, we roughly estimate *δ*R ~ 0.9 *h*.GHz, by fitting the fast Fourier transformed (FFT) spectrum to the calculation result (Fig. 1c, red-dashed curve) derived from a toy-model Hamiltonian^37,39,40^ (see Supplementary Note 2).

The full energy spectrum calculation of the three-electron states using the parameters obtained experimentally across the (2,1)–(1,2) configuration is illustrated in Fig. 1d (right panel). The suppressed *E*_ST_ of the left dot *δL* ~ 19 *h*·GHz is obtained by measuring the width of the EST region in the charge stability diagram with the lever arm of the gate *V*_1_ ~ 0.03. The left QD also allows a weak Wigner molecularization as the measured *δL* is an order of magnitude smaller than the case of non-interacting regime. Because of the small value of *δL*/(*k*_B_*T*_e_) ~ 6, where *k*_B_ is Boltzmann’s constant, thermal tunneling precludes high-fidelity single-shot readout. We obtain data by the time-averaged signal using the correlated-double sampling (CDS) method, which effectively yields the signal proportional to the excited state probability^37^ (see Supplementary Note 3).

**Fig. 2:**
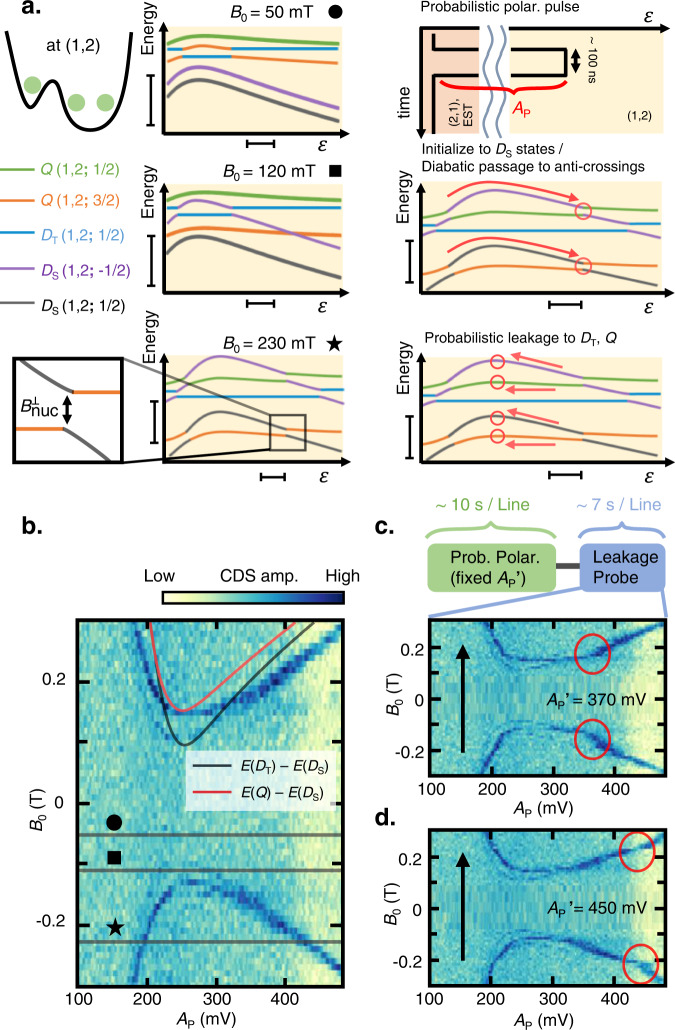
Leakage spectroscopy and probabilistic nuclear polarization with the Wigner molecule. **a** Left panel: schematics of the energy levels for different external magnetic fields *B*_0_ > 0 T. Crossings between different *m*_s_ states become anticrossings aided by the transverse nuclear Overhauser field. Right panel: schematic of the pulse sequence for leakage spectroscopy and probabilistic dynamic nuclear polarization (DNP). The pulse diabatically drives the initialized *D*_S_(2,1;1/2) [*D*_S_(2,1;−1/2)] to (1,2), and hold *ε* for 100 ns ≫ *T*_2_*. Upon the coincidence of the pulse detuning and the anti-crossing, the state probabilistically evolves to *Q*(1,2;3/2) [*Q*(1,2;1/2)] and flips the electron spin Δ*m*_S_ = +1 which accompanies Δ*m*_N_ = −1. The scale bars on the bottom axis (*ε* axis) denote 50 μeV, and the scale bars on the left axis (Energy axis) denote 1 *h*·GHz. **b** Leakage spectroscopy of the Wigner molecule (WM) state as a function of *B*_0_ and the pulse amplitude *A*_p_. Black (Red) dotted curve shows the calculated energy splitting between *D*_T_ (*Q*) and *D*_S_ at *B*_0_ = 0 T. Measurement-induced nuclear field shifts the dispersion opposite to the direction of *B*_0_. **c**, **d** Leakage measurement with an additional probabilistic polarization pulse with amplitude *A*_p_′ applied before each line sweep. The *A*_p_′ is fixed to 370 (450) mV, and the additional distortion in the leakage spectrum is shown as red circles near a pulse amplitude of 370 (450) mV. Black arrows denote the magnetic field sweep direction.

We confirm the WM spin structure via the strongly suppressed energy spectrum in the right QD with varying *B*_0_. We focus on five low-lying energy levels among eight possible multiplet states. See Methods for notations used for labeling spin multiplets. As shown in Fig. 2a (left panel), *D*_S_(1,2;−1/2) [*D*_S_(1,2;1/2)] becomes degenerate with *D*_T_(1,2;1/2) or *Q*(1,2;1/2) [*Q*(1,2;3/2)] at a certain *ε* depending on the *B*_0_ magnitude. The degeneracies are lifted by the transverse Overhauser field ${B}_{{nuc}}^{\perp }$^8,11^. To detect such anticrossings, we first initialize the state to either *D*_S_(2,1;−1/2) or *D*_S_(2,1;1/2) at the EST position. By pulsing the initialized *D*_S_(2,1;−1/2) [*D*_S_(2,1;1/2)] towards (1,2) and holding for ~100 ns ≫ *T*_2_*, mixing with (or leakage to) states *Q*(1,2;1/2) or *D*_T_(1,2;1/2) [*Q*(1,2;3/2)] can occur if the pulse amplitude *A*_p_ coincides with the anti-crossing position (Fig. 2a, right panel). Upon pulsing back to the (2,1) charge configuration, the resultant excited states *Q* or the *D*_T_ probability can be detected via EST^27,37,38^. Figure 2b shows the leakage spectrum versus *A*_P_ and *B*_0_, mapping out the anti-crossing positions similar to “spin-funnel” measurements in two-electron ST_0_ qubits reproducing the energy splittings between the ground and excited levels^8,16,43,44^. The black (red) dashed curves show the calculated splittings (Fig. 1d) between the *D*_S_ and *D*_T_ (*Q*) states at *B*_0_ = 0 T, with the Lande *g*-factor g* ~ −0.4 ^45,46^.

Although the calculated curve qualitatively agrees with the experimental curve, the observed spectrum curvature as a function of *A*_P_ and *B*_0_ is smaller because of the DNP induced by the pulse sequence used for leakage spectroscopy. To confirm this, before each line scan of *A*_p_ in Fig. 2c, d, a similar step pulse with a fixed amplitude *A*_P_′~370 mV (450 mV) is applied for 10 s. Consequently, we observe distortions (red circles) in the spectrum occurring at *A*_P_′. This is because, when *A*_P_′ matches with the anti-crossing position, the pulse probabilistically flips the electron spin with a change in the angular momentum Δ*m*_S_ = +1 by the leakage process described above and accompanies flop Δ*m*_N_ = −1 of the nuclear spin^8,11^. Unlike the electrons in GaAs, nuclei have positive *g*-factors^8,20^; therefore, the Δ*m*_S_ = +1 electron spin flip, and thereby the Δ*m*_N_ = −1 nuclear spin flip polarizes *B*_nuc_ toward the *B*_0_ direction^8,11,47^. This additionally drags the leakage spectrum opposite to the *B*_0_ direction under a specific condition *A*_p_ = *A*_P_’. These results indicate that leakages induced by hyperfine interaction between the WM and nuclear environment lead to an observable change in *B*_nuc_. Despite the long measurement time per line scan (~7 s) owing to the communication latency between the measurement computer and the instruments, the polarization effect is still visible. Thus, *τ*_N_ > 10 s, as discussed below. Moreover, as the anti-crossing position is a sensitive function of *B*_tot_ = *B*_0_ + *B*_nuc_ over 100~300 mT, it can be used to measure *B*_nuc_.

**Fig. 3:**
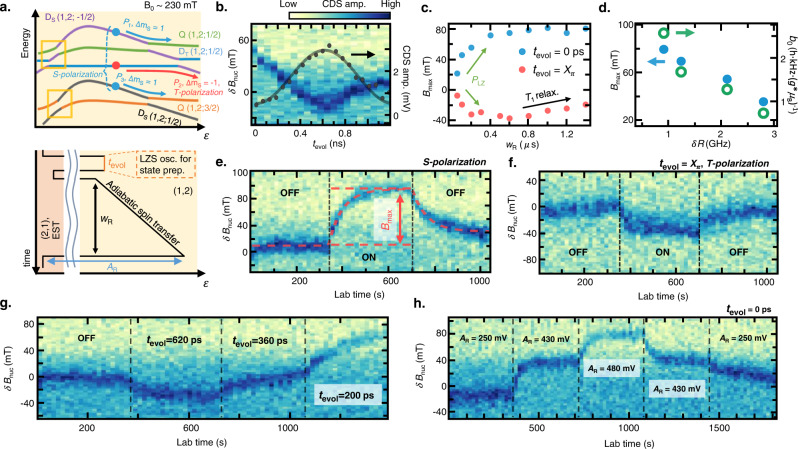
Bidirectional and controllable dynamic nuclear polarization enabled by Wigner molecularization. **a** Top panel: Schematic of the anticrossings used for deterministic dynamic nuclear polarization (DNP). Bottom panel: pulse sequence used for S- and T-polarizations. For *t*_evol_ = 0 ns, the sequence corresponds to maximum S-polarization, which brings *D*_S_(1,2;1/2) [*D*_S_(1,2;−1/2)] adiabatically across the anti-crossing to *Q*(1,2;3/2) [*Q*(1,2;1/2)] flipping the electron spin with Δ*m*_S_ = +1 and leading to Δ*m*_N_ = −1 (blue arrow, S-polarization). For *t*_evol_ = 600 ns, the sequence corresponds to maximum T-polarization. Herein, the *D*_T_(1,2;1/2) prepared with a (Landau–Zener–Stückelberg) LZS-oscillation-induced *π*-pulse is adiabatically transferred to *D*_S_(1,2;1/2), resulting in Δ*m*_S_ = −1 and Δ*m*_N_ = +1 (red arrow, T-polarization), which has the opposite polarization effect compared to S-polarization. **b** Change in the nuclear field *δB*_nuc_ as a function of *t*_evol_. The gray curve shows the corresponding LZS oscillation measurement reflecting the *D*_T_ population. The *δB*_nuc_ oscillates out of phase to the LZS oscillation owing to the oscillation of the S- and T-polarization ratio. **c** The magnitude of the maximum polarization *B*_max_ as a function of ramp time *w*_R_. The *B*_nuc_ saturates to *B*_max_ when the polarization and the nuclear spin diffusion rate reach an equilibrium. For small *w*_R_, the |*B*_max_| decreases because of the small Landau–Zener transition probability *P*_LZ_ for both S- (blue circle) and T-polarizations (red circle). In the case of T-polarization, |*B*_max_| decreases again for long *w*_R_ owing to the lattice relaxation of the excited population. **d** *B*_max_ as a function of *δR*. The polarization gets more efficient for smaller *δR* indicating a strong dependence of the nuclear polarization efficiency on the Wigner parameter. **e, f** Dynamic nuclear control with the S (T)-polarization sequence. The red dotted line is the numerical fit derived from the simple rate equation-based model. The fit yields the nuclear spin diffusion time τ_N_ ~ 62 s, with a polarization magnitude per spin flip of ~2.58 *h*·kHz·(g*μ_B_)^−1^. **g** On-demand DNP via *t*_evol_. **h** Adiabatic ramp amplitude *A*_R_ with *t*_evol_ = 0 ns realizing self-limiting DNP.

We now show bidirectional DNP combined with coherent control of doublet states at *B*_0_ = 230 mT. Figure 3a (top panel) shows the three primary paths through the anticrossings, which can flip the electron spins deterministically by adiabatic passage^2,8,11^. Paths *P*_1_ and *P*_3_ describe the S-polarization that flips the electron spin with Δ*m*_S_ = +1. This is enabled by initializing the state to *D*_S_(1,2;−1/2) [*D*_S_(1,2;1/2)] at the EST position and then by non-adiabatically pulsing beyond the first anticrossings near the (2,1) charge configuration (Fig. 3a, yellow boxes), followed by adiabatically driving the state through the anti-crossing to *Q*(1,2;1/2) [*Q*(1,2;3/2)], which accompanies Δ*m*_N_ = −1 (Fig. 3a, blue arrows). Because the spin state is initialized to the doublet-singlet state before the adiabatic spin-flip passage, the sequence is named S-polarization. The *Q*(1,2;1/2) [*Q*(1,2;3/2)] state is diabatically driven back to the EST position, and one electron quickly tunnels out to the reservoir. Reloading an electron from the reservoir reinitializes one of the *D*s states completing the polarization cycle. Both the *D*_S_(1,2;−1/2) and *D*_S_(1,2;1/2) initial states contribute to the S-polarization. Path *P*_2_ denotes the T-polarization (Δ*m*_S_ = −1, Δ*m*_N_ = +1), which is possible by driving *D*_T_(1,2;1/2) adiabatically to *D*_S_(1,2;−1/2) (Fig. 3a, red arrow). To prepare *D*_T_(1,2;1/2), we apply a π-pulse to *D*_S_(2,1;1/2) before the adiabatic passage (Fig. 3a, bottom panel). Because we prepare the doublet-triplet state before the adiabatic spin-flip passage, the sequence is called T-polarization. The T-polarization is possible only when the state is initialized to *D*_S_(2,1;1/2) at the EST position.

Combining the S- and T-polarizations, we measure the change in *B*_nuc_ (*δB*_nuc_), where the repeated polarization pulse sequence (Fig. 3a, bottom panel) with variable *t*_evol_ and a repetition rate of ~ 20 kHz is applied for 10 s before each line scan. For Fig. 3b, a waiting time ~10 min was added after each sweep to allow the polarized nuclei to diffuse and minimize the polarization effect in the next sweep. As shown in Fig. 3b, *δB*_nuc_ oscillates with *t*_evol_, which is anti-correlated with the LZS oscillation that represents the population of *D*_T_(1,2;1/2). This confirms that the net polarization rates can be controlled by adjusting *t*_evol_. Accordingly, we calibrate *t*_evol_ = 0 (0.62 ns) for S (T)-polarization. We also calibrate the duration of the adiabatic spin transfer *w*_R_. Figure 3c shows the maximum nuclear field change *B*_max_ reachable as a function of *w*_R_, where both S- and T- polarizations are ineffective for short *w*_R_ because of negligible adiabatic transfer probability *P*_LZ_^2,48^. |*B*_max_| reaches a maximum around *w*_R_ ~ 0.8 μs, after which the maximum efficiency is retained for the S-polarization sequence. In the case of T-polarization, however, for long *w*_R_, |*B*_max_| decreases because of *D*_T_ relaxation during the adiabatic passage.

By tuning *δR* via the dc gate voltages and performing similar S-polarization experiments, we find that *B*_max_ decreases with increasing *δR* (Fig. 3d, see Supplementary Note 4). As is discussed subsequently, we find that the nuclear diffusion time scale exceeds 60 s regardless of *δR*, but the Overhauser field change per electron flip *b*_0_ is strongly suppressed with increasing *δR*. Ultimately, the observation implies that the pulsed-gate-based nuclear control becomes inefficient in the non-interacting regime. We suspect the degree of electronic wavefunction localization which depends on the Wigner parameter may be affecting the contact hyperfine interaction between the electron and the nuclear spins and altering the DNP efficiency as a result.

Returning to the condition *δR* ~ 0.9 *h*·GHz, we demonstrate on-demand DNP. Figure 3e, f shows the result of optimized S (T)-polarization with *t*_evol_ = 0 ns, *w*_R_ = 1000 ns (*t*_evol_ = 0.62 ns, *w*_R_ = 600 ns). Although the local fluctuations of the nuclear spins lead to random drift of the anti-crossing positions without the polarization pulse, *B*_nuc_ builds toward (opposite to) the *B*_0_ direction faster than the nuclear spin diffusion timescale when the polarization pulse is applied before each line scan. *δB*_nuc_ rises to *B*_max_ 80 mT (−40 mT) until a dynamic equilibrium is reached. Because only the *m*_s_ = 1/2 states contribute to the T-polarization, |*B*_max_| for the T-polarization is about half of that for the S-polarization, implying that the state initialize to both *m*_s_ states with nearly equal probability at the EST position.

We also demonstrate bidirectional DNP by adjusting *t*_evol_ in Fig. 3g. Figure 3h illustrates the control of *B*_nuc_ by adjusting the adiabatic sweep amplitude *A*_R_ of the S-polarization sequence. Under the S-polarization, *B*_nuc_ builds in the *B*_0_ direction and drives the anti-crossing to deeper *ε* (more to (1,2) charge configuration). Because the pulse cannot have a finite polarization effect if the anti-crossing position is driven beyond *ε* reachable with *A*_R_, *A*_R_ serves as the limiting factor of *B*_max_. Thus, a self-limiting DNP protocol, where the DNP field is limited by experimental parameters used in the pulse shape rather than the interplay between pumping rate and nuclear diffusion, can be realized. This self-limiting property can be useful in future DNP experiments as the steady state DNP field can be simply controlled by adjusting the pulse amplitude.

Using a simple rate equation, we simulate the polarization-probe sequence (red-dashed curve in Fig. 3e, see Methods and Supplementary Note 5) and obtain *τ*_Ν_ ∼ 62 s and *b*_0_ ~ 2.58 *h*·kHz·(g*μ_B_)^−1^ from the fit. In contrast, the DNP effect is negligible in our device with the two-electron ST_0_ qubit^8^ under the same repetition rate as in the WM regime (see Supplementary Note 6). We further find our DNP is still effective when the repetition rate is as low as 5 kHz (see Supplementary Note 7) showing that the Wigner molecule allows sizable DNP that cannot be achieved with conventional QDs. Through optimization of the magnitude and direction of *B*_0_, *b*_0_ ~ 3 h kHz·(g*μ_B_)^−1^ can be achieved with an ST_0_ qubit in GaAs^2,8^. However, the obtained result shows that robust nuclear control can be achieved with WMs even in the regime where the same level of control cannot be achieved with an ST_0_ qubit. In addition, residual polarization ~21.5 mT exists after turning off the polarization sequence (Fig. 3e), which diffuses within ~30 min. The large Knight shift gradient originating from the non-uniformly broadened WM wavefunction may be a possible cause of the long *τ*_Ν_. However, the newly observed phenomena in this study, including the dependence of *b*_0_ on the tuning condition, require further investigations^47,49^.

Furthermore, the WM’s coherent LZS dynamics provide a novel approach to measure the spatial Overhauser field gradient Δ*B*_Z_ between QDs. When Δ*B*_Z_ is larger than the exchange splitting between *D*_T_(1,2;1/2) [*D*_T_(1,2;–1/2)] and *Q*(1,2;1/2) [*Q*(1,2; − 1/2)], the eigenstates are expected to become *D*_T1_(1,2;1/2) = $|\downarrow \rangle|{T}_{+}\rangle$ [*D*_T1_(1,2; − 1/2) = $|\uparrow \rangle|{T}_{-}\rangle$] and *D*_T0_(1,2;1/2) = $|\uparrow \rangle|{T}_{0}\rangle$ [*D*_T0_(1,2; − 1/2) = $|\downarrow \rangle|{T}_{0}\rangle$]^35^. Because both states can tunnel-couple to *D*_S_(1,2;1/2) [*D*_S_(1,2;−1/2)], the LZS oscillation reveals the *D*_T1_ **–** *D*_S_ and *D*_T0_ **–** *D*_S_ energy splittings. As can be inferred from the Hamiltonian (see Supplementary Note 8), although the *D*_T0_ **–** *D*_S_ splitting is independent of the Δ*Β*_Z_ and *B*_Z_, the *D*_T1_ **–** *D*_S_ splitting is modulated by Δ*Β*_Z_ depending on the sign of Δ*Β*_Z_ and *m*_s_, providing the direct measure of Δ*Β*_Z_. Because the states can initialize to both *D*_S_(1,2;1/2) and *D*_S_(1,2;−1/2) at the EST position, the LZS oscillation captures the dynamics of both *m*_s_ = 1/2 and *m*_s_ = −1/2 subspaces.

**Fig. 4:**
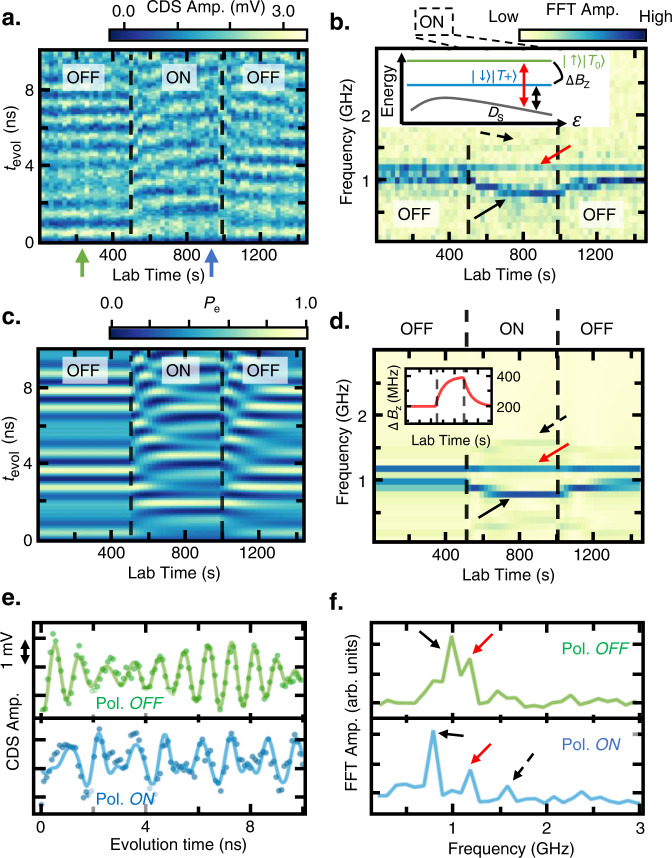
Field gradient control and measurement. Landau–Zener–Stückelberg (LZS) oscillation of the Wigner molecule (WM) states at *B*_0_ = 230 mT in **a** the time domain and **b** the frequency domain with the S-polarization sequence. The oscillation reveals the relative phase oscillation of the *D*_T1_ – *D*_S_ (black arrow, black dotted arrow) and *D*_T0_ – *D*_S_ (red arrow) of both the *m*_s_ = 1/2 and *m*_s_ = −1/2 states. The *D*_T0_ – *D*_S_ splitting is constant regardless of the magnetic field gradient Δ*B*_Z,_ whereas the *D*_T1_ – *D*_S_ energy spacing is modulated by the Δ*B*_Z_ depending on the sign of Δ*B*_Z_ and *m*_s_. The resultant beating is visible in **e**, **f** the time (frequency) domain line-cut when the polarization is on (green arrow in **a**) and off (blue arrow in **a**). The line cuts in the time domain are numerically fitted to the sum of three sine functions (solid lines in **e**) with different amplitudes. Three separate peaks are visible in the frequency domain (**f**) when the Δ*B*_Z_ is largely polarized in the bottom panel (blue line) in (**f**) Simulated LZS oscillation in **c** the time domain and **d** the frequency domain with the Δ*B*_Z_ in the inset of (**d**). The simulation in the frequency domain reproduces the branches shown in (**b**).

Figure 4a (4b) illustrates the LZS oscillation measurement of the WM multiplet states at *B*_0_ = 230 mT in the time (frequency) domain with the S-polarization turned on and off at specific laboratory times. The FFT spectrum exhibits three different branches corresponding to the *D*_T0_ – *D*_S_ (red arrow) and *D*_T1_ – *D*_S_ (black and black-dashed arrows) where the beating patterns vary as the S-polarization induces changes in Δ*B*_z_. Two different *D*_T1_ – *D*_S_ branches correspond to different *m*_s_ subspaces, where the sign of Δ*B*_z_ should be known to distinguish the *m*_s_ for each branch. The *D*_T0_ – *D*_S_ splitting is the same for both *m*_s_ subspaces and is displayed as a single branch (red arrow). Figure 4c, d shows the simulated time (frequency) domain signal of the same LZS oscillation, which agrees well with the experimental result (see Supplementary Note 9). As expected, the *D*_T0_ – *D*_S_ splitting is constant regardless of Δ*B*_z_, whereas the *D*_T1_ – *D*_S_ splitting is modulated along the polarization sequence.

The *D*_T0_ – *D*_T1_ splitting without the polarization sequence implies the built-in Δ*B*_z_ ~ 200 *h*·MHz·(g*μ_B_)^−1^ (35 mT), which is also confirmed by the ST_0_ oscillation (see Supplementary Note 6). Δ*B*_z_ increases to 400 *h*·MHz·(g*μ_B_)^−1^ (70 mT) with the S-polarization and decreases to 200 h MHz·(g*μ_B_)^−1^ after turning the polarization off. Thus, we conclude that the S-polarization yields the asymmetric pumping effect (Δ*B*_nuc_ ~ 200 h MHz·(g*μ_B_)^−1^) about the QD sites, whereas the Δ*B*_nuc_ direction can be experimentally checked, for example, via single-spin electric-dipole spin resonances^46^. Furthermore, the *D*_T0_ – *D*_S_ splitting comprises the decoherence-free subspace for the qubit operations resilient to magnetic noises, where the coherent microwave control combined with the large polarization may enable leakage-free and state-selective transitions.

## Discussion

The present work uncovers the spin and energy structure of the WM states and explores the central-spin problem with strongly correlated WM states in semiconductor QDs. With the energy splitting of the WM ~ 0.9 *h*·GHz, we confirm the controllable DNP of *B*_nuc_ (Δ*B*_nuc_) reaching (but not limited to) 80 mT (35 mT) via leakage spectroscopy and LZS oscillations. The *τ*_N_ exceeds 60 s, which, together with bidirectional polarizability, is beneficial for stabilizing the nuclear bath fluctuation and realizing long-lived nuclear polarization^10,15^.

We anticipate several directions for further developments and applications of WM-enabled DNP. Similar experiments with a larger *δL/T*_e_ ratio can enable high-fidelity single-shot readout for a faster measurement of the dynamics of nuclear polarization. This would further enable feedback loop control^10^ and tracking^12,50,51^ of nuclear environments in multielectron QDs which can be utilized to narrow the nuclear field distribution for electron coherence enhancement. The real-time Hamiltonian estimation also improves frequency resolution for measuring instantaneous Δ*B*_nuc_, which may enable measurements of the degree of spatial localization within WMs. We expect more asymmetric QD geometry would allow a smaller energy gap and thereby the broader electronic wavefunction. Along with the tunability of the energy gap via the gate voltage, as shown here, this may facilitate the investigation of the spatial noise characteristics within a single QD which has been impractical with the typical QD geometries. Furthermore, DNP becomes inefficient with increasing *E*_ST_ of the WM, as discovered herein. This implies that the pulsed-gated electron–nuclear flip-flop probability is a strong function of the Wigner parameter, the microscopic origin of which requires more rigorous investigations.

## Methods

### Device fabrication

A quadruple QD device was fabricated on a GaAs/AlGaAs heterostructure with a 2DEG formed ~70 nm below the surface. The transport property of the 2DEG showed mobility *μ* = 2.6 × 10^6^ cm^2^(V s)^−1^ with electron density *n* = 4.0 × 10^11^ cm^−2^ at temperature *T* = 4 K. Electronic mesa around the QD site was defined by the wet etching technique, and thermal diffusion of a metallic stack of Ni/Ge/Au was used to form the ohmic contacts. The depletion gates were deposited on the surface using standard e-beam lithography and metal evaporation of 5 nm Ti/30 nm Au. The lithographical width of the inner QD along the QD axis direction was designed to be ~10% wider than the outer dot to facilitate WM formation. The QD array was aligned to the [110] crystal axis, as shown in Fig. 1a. Although the magnetic field *B*_0_ was intended to be applied perpendicular to the [110] axis to minimize the effect of spin-orbit interaction^2^, the angular deviation was not strictly calibrated.

### Measurement

The device was placed on a ~40 mK plate in a commercial dilution refrigerator (Oxford instruments, Triton-500). Ultra-stable dc-voltages were generated by battery-powered dc-sources (Stanford Research Systems, SIM928). They were then combined with rapid voltage pulses from an arbitrary waveform generator (AWG, Keysight M8195A with a sample rate up to 65 GSa/s) via homemade wideband (10^1^–10^10^ Hz) bias tees to be applied to the metallic gate electrodes. An LC-tank circuit with a resonant radio frequency (rf) of ~120 MHz was attached to the ohmic contact near the SET charge sensor to enable high-bandwidth (*f*_BW_ > 1 MHz) charge detection^27,31–33,37^. The reflected rf-signal was first amplified by 50 dB using two-stage low-noise cryo-amplifiers (Caltech Microwave Research, CITLF2 ×2 in series) at a 4 K plate. Next, it was further amplified by 25 dB at room temperature using a homemade low-noise rf-amplifier. The signal was then demodulated by an ultra-high-frequency lock-in amplifier (Zurich Instruments, UHFLI), which was routed to the boxcar integrator built in the UHFLI. Trigger signals with a repetition period of 51 μs were generated by a field-programmable-gate array (FPGA, Digilent, Zedboard) to synchronize the timing of the AWG and the boxcar integrator for the CDS^37^.

### Eigenstates of three-electron spin states

Three-electron spin-multiplet structure consists of eight different eigenstates, which are two *D*_S_ states, two *D*_T_ states, and four quadruplet states For simplicity, we show only the spin states with (1,2) charge configuration in Table 1^34–36^, where the spin state in the first (second) bracket indicates the single- (two-) electron spin state in the left (right) QD.

**Table 1:** Three-electron spin states

State	Spin structure
*Q*(1,2; *m*_s_ = 3/2)	$|\uparrow \rangle|{T}_{+} \rangle$
*Q*(1,2; *m*_s_ = 1/2)	$\frac{1}{\sqrt{3}} [\sqrt {2}|\uparrow \rangle|{T}_{0}\rangle+|\downarrow \rangle|{T}_{+}\rangle ]$
*Q*(1,2; *m*_s_ = −1/2)	$\frac{1}{\sqrt{3}}[\sqrt{2}|\downarrow \rangle|{T}_{0}\rangle+|\uparrow \rangle|{T}_{-}\rangle ]$
*Q*(1,2; *m*_s_ = −3/2)	$|\downarrow \rangle|{T}_{-}\rangle$
*D*_S_(1,2; *m*_s_ = 1/2)	$|\uparrow \rangle|S\rangle$
*D*_T_(1,2; *m*_s_ = 1/2)	$\frac{1}{\sqrt{3}}[|\uparrow \rangle|{T}_{0}\rangle -\sqrt{2}|\downarrow \rangle|{T}_{+}\rangle ]$
*D*_S_(1,2; *m*_s_ = −1/2)	$|\downarrow \rangle|S\rangle$
*D*_T_(1,2; *m*_s_ = −1/2)	$\frac{1}{\sqrt{3}}[|\downarrow \rangle|{T}_{0}\rangle -\sqrt{2}|\uparrow \rangle|{T}_{-}\rangle ]$

Here, *T*_0_, *T*_+_ and *T*_−_ denote the three triplet states (*S* = 1) with *m*_s_ = 0, +1 and −1 respectively and S indicates the spin singlet state (*S* = 0).

### Rate equation

Nuclear spin polarization and the diffusion process were phenomenologically modeled using a rate equation:

1 $$\frac{d{{B}}_{{{{{\rm{nuc}}}}}}}{d{t}}=-{{B}}_{{{{{\rm{nuc}}}}}}/{\tau }_{N}+{{b}}_{0}{{P}}_{{{{{\rm{flip}}}}}}/{{T}}_{{{{{\rm{rep}}}}}},$$

where *τ*_N_ is the nuclear spin diffusion time, *b*_0_ is the Overhauser field change per electron spin-flip, *P*_flip_ is the nuclear spin flop probability obtained from the Landau–Zener transition probability *P*_LZ_ and the false initialization probability (see Supplementary Note 5), and *T*_rep_ is the pulse repetition period. Using Eq. (1), we simulated the polarization-probe sequence shown in Fig. 3 with the experimental parameters including the time required for the amplitude sweep in the leakage probe step.

## Supplementary information

Supplementary Information

## Data Availability

The data that support the findings of this study are available from the corresponding author upon request.
