# Supplementary material for: Wigner-molecularization-enabled dynamic nuclear polarization
Source: Nat Commun. 2023 May 23;14:2948. doi: 10.1038/s41467-023-38649-5 (PMC10205738; doi:10.1038/s41467-023-38649-5)
Supplement: Supplementary file 1 — Supplementary Information [file 41467_2023_38649_MOESM1_ESM.pdf]

Wigner-molecularization-enabled dynamic nuclear polarization

Wonjin Jang[1], Jehyun Kim[1], Jaemin Park[1], Gyeonghun Kim[1], Min-Kyun Cho[1], Hyeongyu Jang[1], Sangwoo Sim[1], Byoungwoo Kang[1], Hwanchul Jung[2], Vladimir Umansky[3], and Dohun Kim[1]*

[1]*Department of Physics and Astronomy, and Institute of Applied Physics, Seoul National University, Seoul 08826, Korea*

[2] *Department of Physics, Pusan National University, Busan 46241, Korea*

[3]*Braun Center for Submicron Research, Department of Condensed Matter Physics, Weizmann Institute of Science, Rehovot 76100, Israel*

**Corresponding author: dohunkim@snu.ac.kr*

Supplementary Information

Supplementary Note 1. Electron temperature

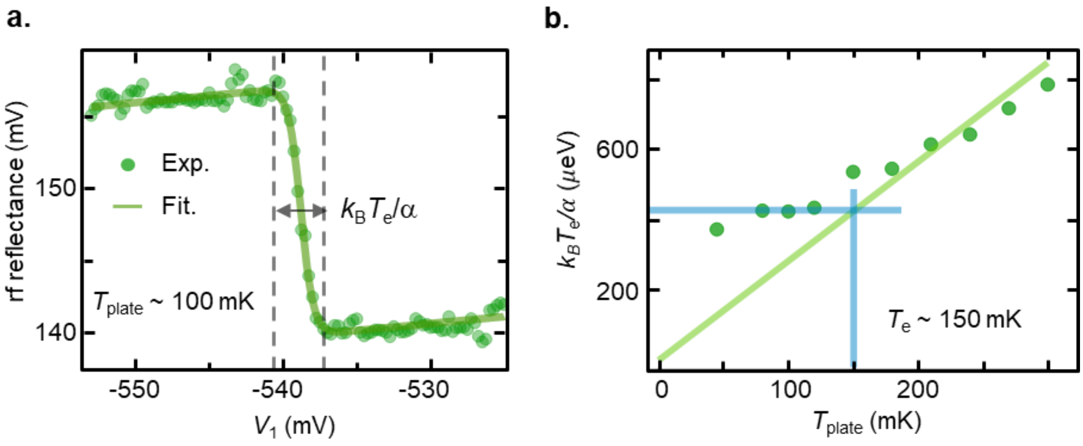

Supplementary Figure 1. Electron temperature measurement. a. Charge transition line broadening due to the finite electron temperature. The radio-frequency (rf)-single-electron transistor (rf-SET) charge sensing signal is recorded as a function of the gate voltage V_1 near the (2,1)–(1,1) charge transition at the mixing chamber plate temperature of the dilution refrigerator T_{plate} ~ 100 mK. The solid curve is a fit to the Fermi–Dirac distribution with a linear background slope, from which we obtain the thermal broadening $k_B T_e/\alpha$, where k_B is the Boltzmann constant, T_e is the electron temperature, and α is the lever arm of V_1. **b.** $k_B T_e/\alpha$ measured with varying T_{plate}. From the linear relationship for T_{plate} > 200 mK and plateau for T_{mixing} < 100 mK, we estimate T_e = 150 mK and $\alpha = 0.03$, respectively.

Supplementary Note 2. Electronic Hamiltonian

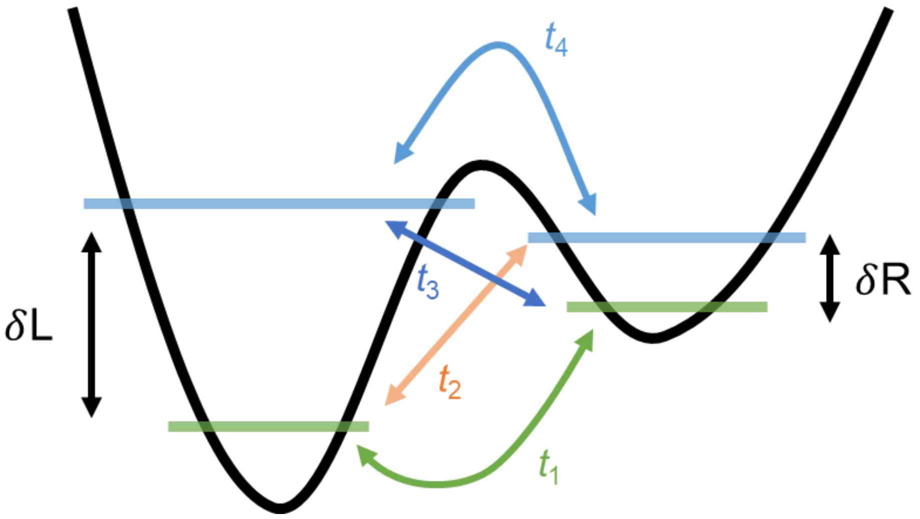

Supplementary Figure 2. Schematic of the three-electron energy levels in a double quantum dot. Orbital level denoted in green (blue) line is the ground (excited) orbital level in each QD. δL (δR) is the orbital splitting in the left (right) QD. t_i (i = 1, 2, 3 and 4) indicates the tunnel coupling between different orbitals.

As mentioned in the main text, in the (1,2) [(2,1)] charge configuration, if the two electrons in the right [left] QD form a spin singlet state, the two electrons occupy the ground orbital in the right [left] QD (green orbital level in Supplementary Fig. 2). The resulting three-electron spin state is the doublet-singlet state $D_S(1,2)$ [$D_S(2,1)$]. When the two electrons form the spin triplet state, the two electrons occupy the excited orbital in the right [left] QD (blue orbital level in Supplementary Fig. 2), where the resulting three-electron state form the doublet-triplet $D_T(1,2)$ [$D_T(2,1)$] or the quadruplet $Q(1,2)$ [$Q(2,1)$] state depending on the specific spin configuration.

When B_0 = 0 T, the D_S states, D_T states, and Q states are degenerate respectively, resulting in three different branches in the energy dispersion. We use a simple toy-model Hamiltonian adopted from the double QD hybrid qubit[1,2].Because there are (2,1) and (1,2) charge configurations available for the each spin state D_S, D_T, and Q, a 6 × 6 Hamiltonian can

describe the dynamics. The ordered basis for the Hamiltonian is [$D_S(2,1)$, $D_T(2,1)$, $Q(2,1)$, $D_S(1,2)$, $D_T(1,2)$, $Q(1,2)$], where n (m) denotes the number of electrons in the left (right) QD by (n, m). We omit the m_S here due to the degeneracy when $B_0 = 0$ T.

$$H_{\mathrm{elec}} = \begin{bmatrix} \varepsilon/2 & 0 & 0 & t_1 & -t_2 & 0 \\ 0 & \varepsilon/2 + \delta\mathrm{L} & 0 & -t_3 & t_4 & 0 \\ 0 & 0 & \varepsilon/2 + \delta\mathrm{L} & 0 & 0 & t_4 \\ t_1 & -t_3 & 0 & -\kappa\varepsilon/2 + \delta\mathrm{R} & 0 & 0 \\ -t_2 & t_4 & 0 & 0 & -\varepsilon/2 + \delta\mathrm{R} & 0 \\ 0 & 0 & t_4 & 0 & 0 & -\varepsilon/2 + \delta\mathrm{R} \end{bmatrix} \quad (1)$$

Here, ε is the energy detuning between the double QD, t_i is the tunnel coupling strength between different orbitals (i = 1, 2, 3, 4) (Supplementary Fig. 2), and δL (δR) is the orbital energy splitting in the left (right) dot. Further, κ is a factor to account for the different lever-arms of the ground and excited states in the (1,2) WM states[3], recently shown to be the consequence of many-body effects[4,5]. The Hamiltonian is utilized to obtain the energy spectra shown in Fig. 1 along ε. As we discuss in detail in Supplementary Note 9, the LZS oscillation at non-zero B_0 is simulated by adding the hyperfine interaction terms[6,7] to the aforementioned Hamiltonian and by solving the time-dependent Schrodinger equation with the experimentally obtained parameters.

Supplementary Note 3. Correlated double sampling (CDS)

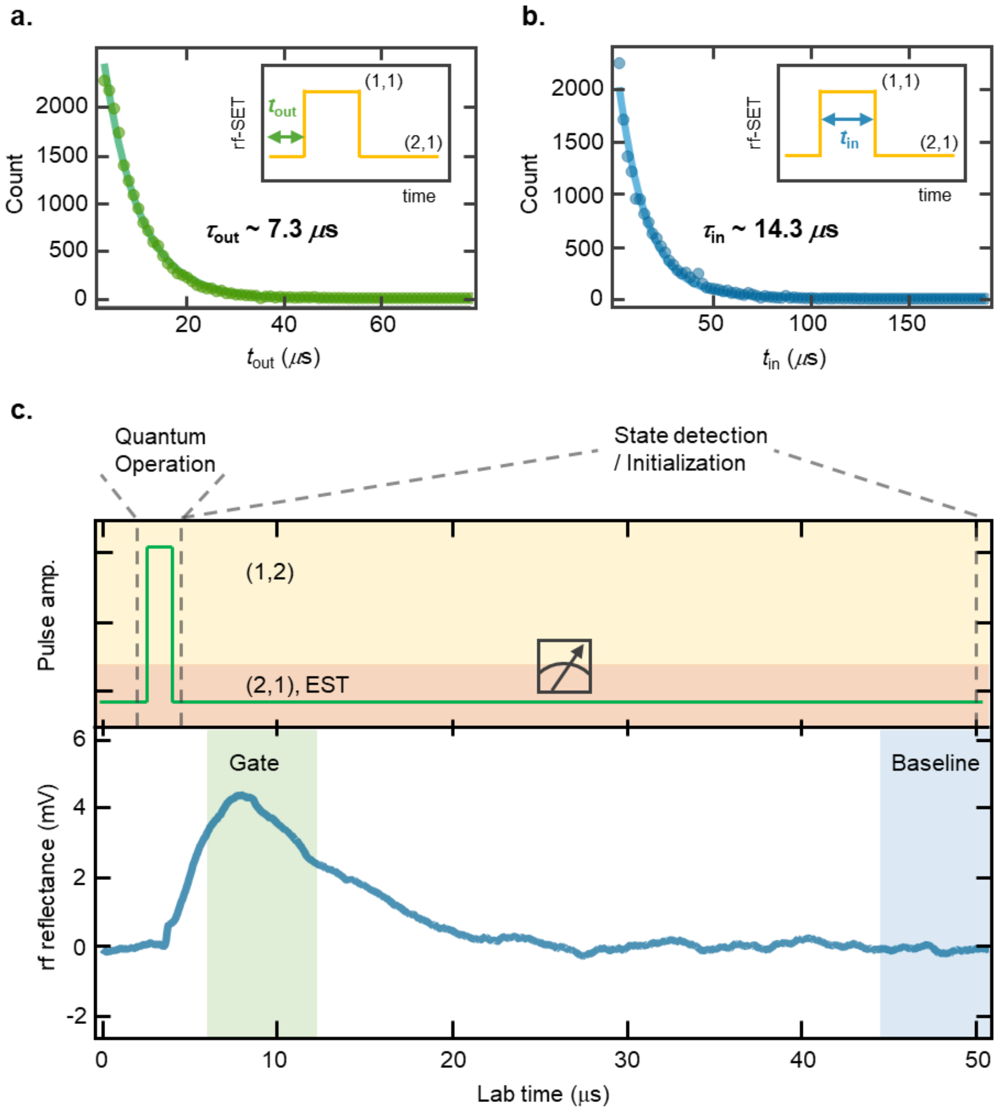

Supplementary Figure 3. Tunneling time scale and correlated double sampling (CDS). a. (b.) Tunneling-out (-in) statistics. The solid curve is a fit to an exponential decay yielding the tunneling-out (-in) time τ_{out} (τ_{in}) ~ 7 (14) μs. Inset in each figure shows a schematic of the charge sensor signal showing tunneling events in the (2,1) energy-selective tunneling (EST) region[8–11] recorded with the radio-frequency (rf)-single-electron transistor (rf-SET). **c.** Top panel: schematic of the quantum control sequence. The pulse brings the initialized state from (2,1) to the operation point in (1,2) and drives back to (2,1) for the EST readout and state initialization. Bottom panel: periodically averaged (~10^6 lines) ac-coupled rf-SET signal synchronized with the Landau–Zener–Stückelberg (LZS)-induced X_π pulse. The dc-offset-eliminated CDS amplitude is generated by subtracting the baseline signal (blue shaded box) from the gate signal (green shaded box) and averaging ~10^3 times via the boxcar integrator. As discussed in the main text, the boxcar integration and the control waveform generation is synchronized to a trigger signal with period of 51 μs.

Supplementary Note 4. Dynamic nuclear polarization under different tunings of Wigner molecule energy spectrum

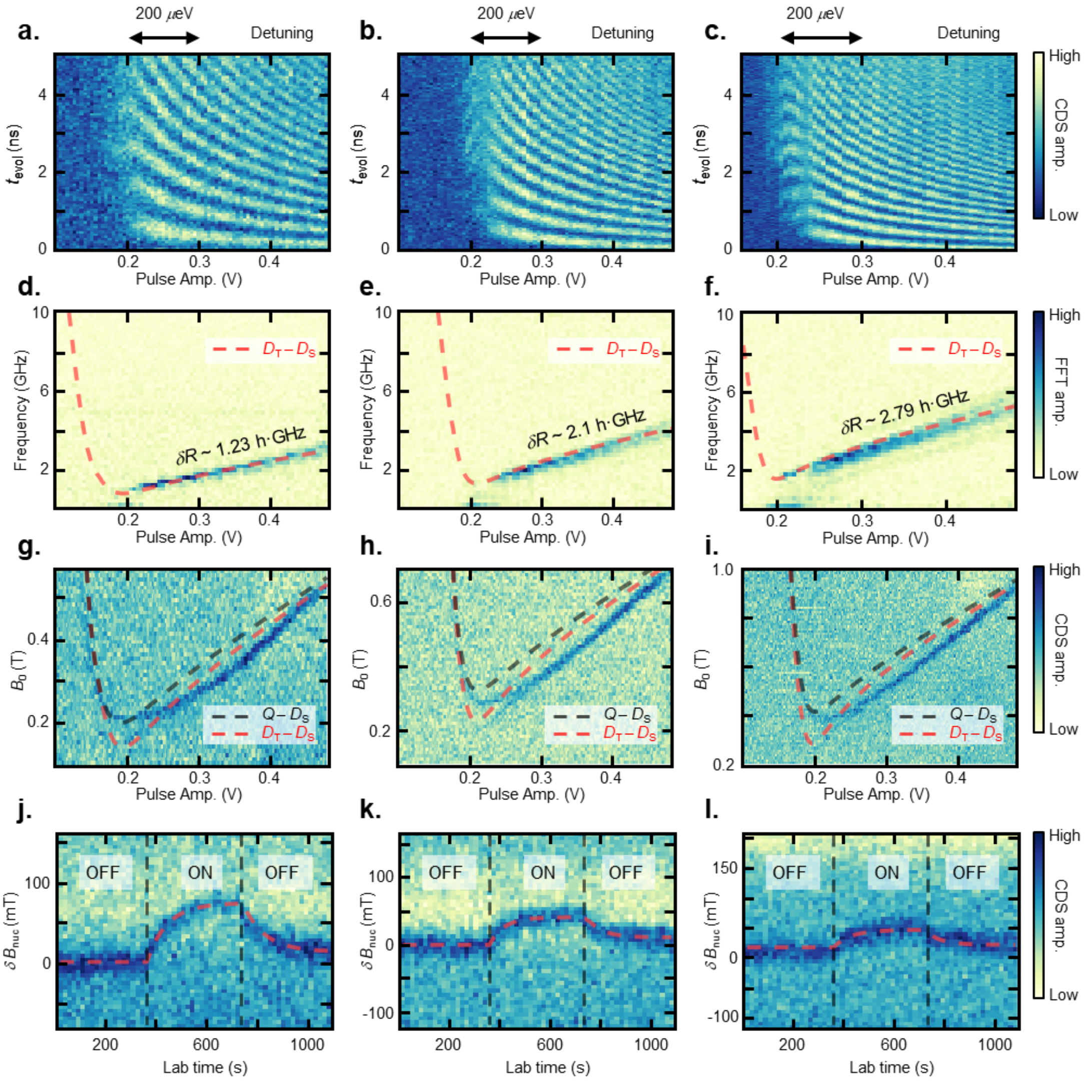

Supplementary Figure 4. Dynamic nuclear polarization under different tunings of Wigner molecule energy spectrum. Time (frequency) domain Landau–Zener–Stuckelberg oscillation with the singlet-triplet splitting of the Wigner molecule (WM), δR of **a.** (**d.**) ~ 1.23 h·GHz, **b.** (**e.**) 2.1 h·GHz, and **c.** (**f.**) 2.79 h·GHz. Red-dashed curves in the frequency domain signals (**d.**, **e.**, and **f.**) show the energy splitting between D_T and D_S states derived from the toy-model Hamiltonian (see Methods section), from which we extract the magnitude of δR. The δR is tuned with the dc-gate-voltages. Leakage spectroscopy of the WM with δR of **g.** ~ 1.23 h ·GHz, **h.** 2.1 h·GHz, and **i.** 2.79 h·GHz. Red (black) dashed curves are the $D_T - D_S$ ($Q - D_S$) energy spacings calculated from the toy-model Hamiltonian with the Lande g-factor $g^* \sim -0.4$. δB_{nuc} measurement with the S-polarization turned on and off with δR of **j.** ~ 1.23 h·GHz, **k.** 2.1 h·GHz, and **l.** 2.79 h·GHz. Although the nuclear spin diffusion time is τ_N ~ 60 s for all tuning, the nuclear polarization strength per electron spin flip b_0 decreases with increasing δR, as shown in Fig. 3d in the main text, resulting in smaller B_{max} for larger δR (i.e., smaller Wigner

parameter)

Supplementary Note 5. Numerical simulation of the nuclear polarization sequence

The nuclear field B_{nuc} during the dynamic nuclear polarization (DNP) is numerically reproduced using the rate equation as follows:

$$\frac{\mathrm{d}B_{\mathrm{nuc}}}{\mathrm{d}t} = -B_{\mathrm{nuc}}/\tau_{\mathrm{N}} + b_0 P_{\mathrm{flip}}/T_{\mathrm{rep}} \,. \qquad (2)$$

As discussed in the Methods section, τ_{N} is the nuclear spin diffusion time, T_{rep} is the repetition period of the polarization pulse, b_0 is the change in the nuclear field per electron spin flip, and P_{flip} is the spin-flip probability obtained from the Landau–Zener transition probability P_{LZ} and the false initialization probabilities β.

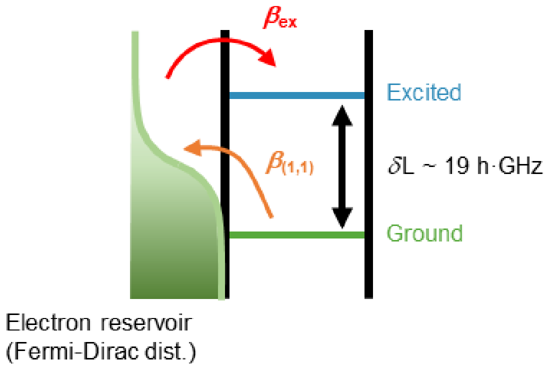

Supplementary Figure 5. Schematic of false initialization at energy-selective tunneling. False initialization to the (1,1) ($\beta_{(1,1)}$, orange arrow) or the excited orbitals (β_{ex}, red arrow) may occur owing to thermal tunneling.

We first analyze the spin-flip probability P_{flip} per adiabatic passage. Because of the small singlet-triplet splitting in the (2,1) EST region δL ~ 19 h·GHz, where h is Planck's constant, the false initialization probability to (1,1) at the start of the pulse is $\beta_{(1,1)}$ ~ 0.37

(Supplementary Fig. 5, orange arrow), which does not contribute to the polarization. We also estimate the probability of the false initialization to the excited orbitals β_{ex} from the Fermi–Dirac distribution with $T_e \sim 150$ mK, as described in Supplementary Note 1. With the Fermi level of the reservoir straddling in the middle of the $D_T - D_S$ splitting, we find $(\beta_{ex})^{-1} \sim Z = 1 + \exp(\delta L/2)/k_B T_e) = (0.049)^{-1}$, where Z is the partition function[12]. Because the falsely initialized state in the excited orbital contributes to the polarization in the opposite direction, we calculate $P_{flip} = P_{LZ} \cdot (1 - \beta_{(1,1)} - 2\beta_{ex})$. We estimate $P_{LZ} \sim 0.5$ for the given adiabatic ramp width w_R from Fig. 3, as the maximum efficiency is saturated for $w_R > 0.8$ μs. The resultant P_{flip} is 0.26.

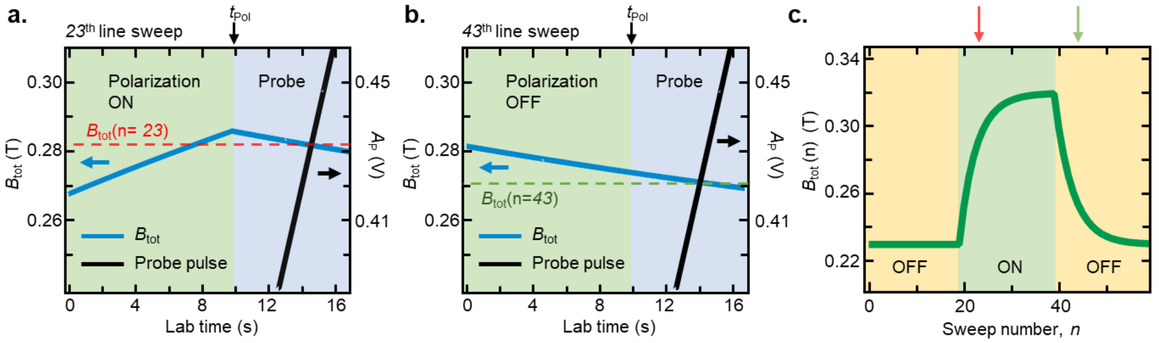

Supplementary Figure 6. a. Schematic of the net magnetic field $B_{tot} = B_0 + B_{nuc}$ during the polarization and the probe stages. During the polarization stage (green shaded area), B_{nuc} builds up and then decays at the probe stage (blue shaded area) owing to nuclear diffusion. The decaying B_{tot} is probed by the pulse amplitude sweep denoted by the black solid line. The crossing of B_{tot} and the pulse amplitude is recorded as the leakage point (red-dotted line in **a.**, red arrow in **c.**). **b.** B_{tot} with the polarization turned off. The crossing point (green-dotted line) is recorded as B_{tot} (n = 43) shown in **c.** (green arrow). **c.** Simulation of B_{tot} during the S-polarization sequence. When the polarization is turned on, B_{tot} builds in the direction of the B_0 and then decays back when the polarization is turned off.

To simulate the polarization sequence, we consider the duration of the polarization stage $t_{Pol} \sim 10$ s and the adiabatic ramp amplitude A_R by setting the P_{flip} to 0.26 only if the laboratory time $t_{lab} < t_{Pol}$ and $B_{tot} = B_0 + B_{nuc} < B_L(A_R)$; otherwise, we set $P_{flip} = 0$. Here, B_L is the one-to-one function between the pulse amplitude and the magnetic field strength obtained

from the leakage spectrum in Fig. 2b. This reproduces the experimental situation where the polarization pulse is turned on for t_{Pol} only when the anti-crossing is reachable with the maximum pulse amplitude, as shown in the green-shaded area in Supplementary Fig. 6a. We convert the number of polarized nuclei to B_{nuc} via b_0.

Based on the above-mentioned setting, we numerically mimic the leakage measurement shown in Fig. 3e. We check for the point where the crossing of the decaying B_{tot} and the probe pulse amplitude (black line in Supplementary Fig. 6a, 6b) occurs at the probe stage (Supplementary Fig. 6a, (6b), red (green) dashed line) and denote it as $B_{tot}(n)$ for the nth leakage measurement line sweep. Supplementary Fig. 6c shows a collection of crossing points $B_{tot}(n)$ with the polarization turned on and off along n, which reflects the leakage measurement with the polarization sequence turned on and off, respectively. We fit $B_{tot}(n)$ to the leakage measurement in Fig. 3e and obtain $b_0 \sim 2.58\ h \cdot \mathrm{kHz} \cdot (g^* \mu_B)^{-1}$ and $\tau_N \sim 62$ s.

Supplementary Note 6. Inefficient nuclear polarization in the two-electron singlet-triplet qubit regime

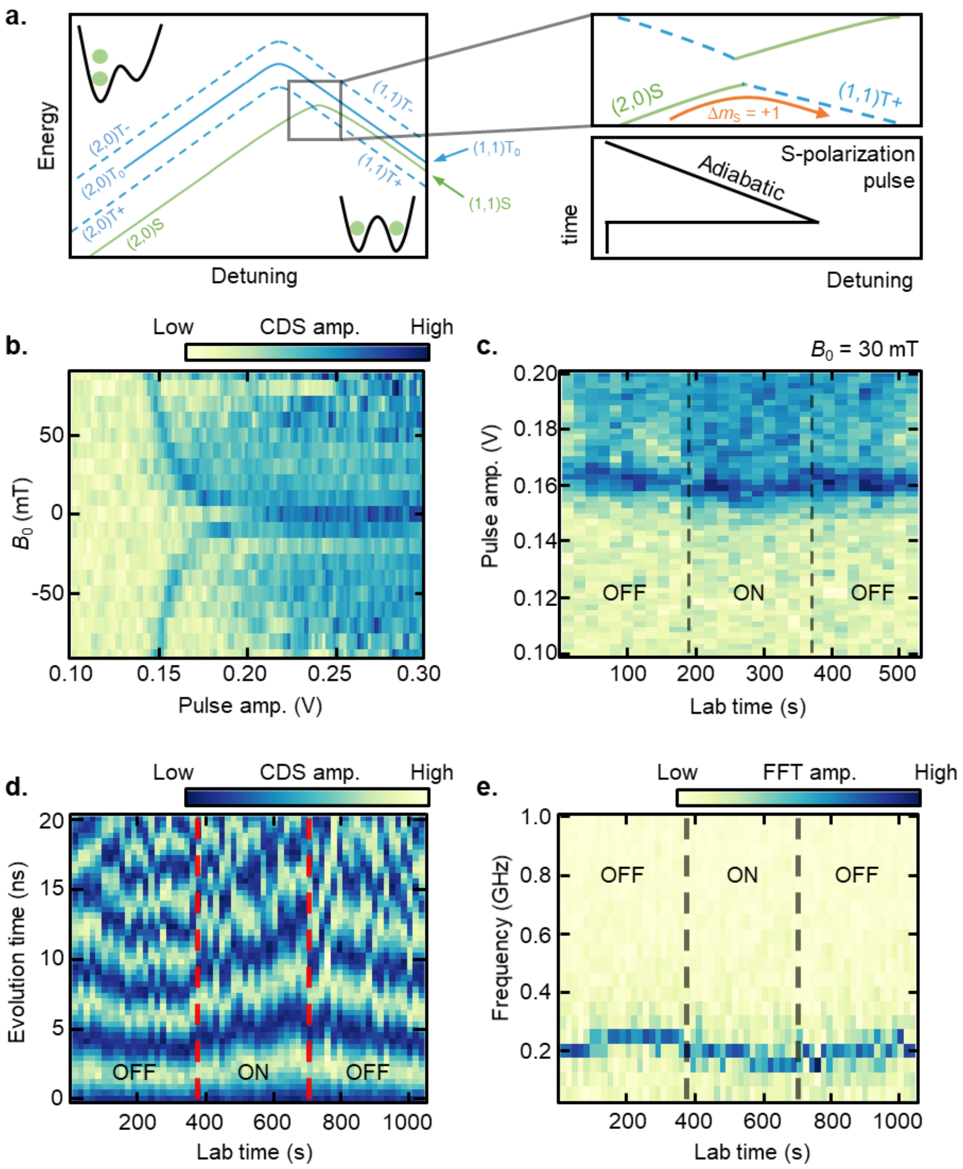

Supplementary Figure 7. a. Left panel: schematic of the singlet-triplet (ST_0) qubit energy levels in the two-electron regime. Zeeman-split T+ level crosses with the singlet branch (black rectangle) resulting in the Overhauser field-mediated anti-crossing. Right panel: magnified view of the anti-crossing with the pulse sequence shown below for the S-polarization (Δm_S = +1, $\Delta m_N = -1$) with the ST_0 qubit[13,14]. **b.** Leakage spectroscopy (spin-funnel) of the singlet-triplet (ST_0) qubit. The spectrum reveals the S–T+ anti-crossing points as a function of B_0. **c.** Leakage measurement at B_0 = 30 mT with the S-polarization turned on and off with a pulse repetition period of 51 μs. No significant signature of B_{nuc} exceeding the fluctuation was found. **d. (e.)** Time (frequency) domain signal of the ST_0 qubit Larmor oscillation at B_0 = 230 mT with

the S-polarization turned on and off. A built-in $|\Delta B_Z| = |B_Z^L - B_Z^R| \sim 200\ h\cdot\text{MHz}\cdot(g^*\mu_B)^{-1}$ exists, where the additional polarization effect is not significantly larger than the fluctuation.

In this section, we show the two-electron singlet-triplet (ST_0) spin qubit operation to compare the nuclear polarization effect in the same device. Supplementary Fig. 7a shows typical two-electron energy levels in a double quantum dot (QD)[15]. The Zeeman-split T+ level crosses with the singlet branch, and the crossing becomes an anti-crossing aided by the finite transverse nuclear Overhauser field[13,14] (right panel in Supplementary Fig. 7a).

Utilizing the EST readout in the (2,0) charge configuration[10], we first measure the leakage spectrum of the ST_0 qubit by probing the S–T+ anti-crossings as a function of B_0 (Supplementary Fig. 7b). Because the leakage position is sensitive to the magnetic field only for $|B_0| < 50$ mT, we set $B_0 = 30$ mT and investigate the effect of S-polarization in Supplementary Fig. 7c. We use the same $T_{\text{rep}} \sim 51\ \mu$s as described in the main text and measure the anti-crossing position with the polarization pulse turned on and off with the same polarization-probe sequence shown in Fig. 3e. As a result, we find that the polarization effect is found to not be as significant as in the Wigner molecule (WM) case shown in Fig. 3. This is consistent with a previous report[13], where a sizable B_{nuc} is only observable for $T_{\text{rep}} < 30\ \mu$s using ST_0 qubit.

The Larmor oscillation frequency of the ST_0 qubit corresponds to the size of the spatial magnetic field gradient ΔB_Z between the double QD (DQD)[15,16]. We also measure the ST_0 Larmor oscillation with the S-polarization turned on and off at $B_0 = 230$ mT, as shown in Supplementary Fig. 7d, with the same sequence as in Fig. 4. We first note that there exists a built-in $\Delta B_Z \sim 200\ h\cdot\text{MHz}\cdot(g^*\mu_B)^{-1}$ stemming from the nuclear Overhauser field, consistent with the energy splitting between the D_{T1} and D_{T0} energy levels without the polarization, as

shown in Fig. 4b and 4d. In contrast to the WM case shown in the main text where the S-polarization yields a change of $|\Delta B_Z| = |B_L^Z - B_R^Z| \sim 200\ h{\cdot}\mathrm{MHz}{\cdot}(g^*\mu_B)^{-1}$, the S-polarization with the ST_0 qubit does not induce a polarization that is significantly larger than the nuclear field fluctuation with the same $T_{rep} \sim 51\ \mu s$ [[13]]. This indicates that a large Knight field shift aided by the non-uniform broadening of the WM wavefunction may suppress the nuclear spin diffusion and lead to sizable nuclear polarization despite the slow pulse repetition rate[17,18].

Supplementary Note 7. Dynamic nuclear polarization under slow pumping rate

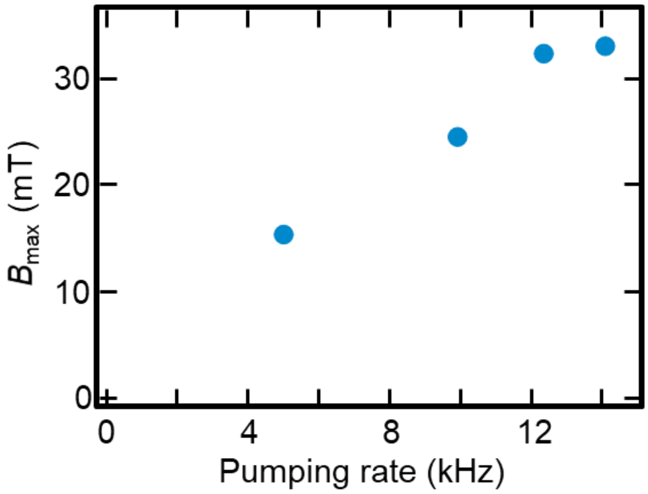

Supplementary Figure 8. Maximum DNP magnitude along the pumping rate. DNP with the WM yields a sizable polarization effect down to ~ 5 kHz pumping rate. The data is acquired in an unideal tuning which differs from that of Fig. 3 in the main text

Supplementary Note 8. Magnetic Hamiltonian

We adopt the hyperfine Hamiltonian from the exchange-only qubit defined in a triple QD[7] as follows. The ordered basis for the Hamiltonian is [D_S(1,2;1/2), D_T(1,2;1/2), Q(1,2;1/2), Q(1,2;3/2), D_S(1,2;−1/2), D_T(1,2;−1/2), Q(1,2;−1/2), Q(1,2;−3/2)] using the (n, m; S_Z) notation introduced in the main text. The Hamiltonian is also identical for the (2,1) charge states.

$$H_{hf}=\left[\begin{array}{cccc|cccc}
\frac{1}{2}B^{Z}_{100} & -\frac{1}{2\sqrt{3}}B^{Z}_{01\bar{1}} & -\frac{1}{\sqrt{6}}B^{Z}_{01\bar{1}} & \frac{1}{2\sqrt{2}}B^{+}_{01\bar{1}} & -\frac{1}{2}B^{-}_{100} & \frac{1}{2\sqrt{3}}B^{+}_{01\bar{1}} & -\frac{1}{2\sqrt{6}}B^{-}_{01\bar{1}} & 0 \\
-\frac{1}{2\sqrt{3}}B^{Z}_{01\bar{1}} & \frac{1}{6}B^{Z}_{\bar{1}22} & -\frac{1}{3\sqrt{2}}B^{Z}_{\bar{2}11} & \frac{1}{2\sqrt{6}}B^{+}_{\bar{2}11} & \frac{1}{2\sqrt{3}}B^{-}_{01\bar{1}} & \frac{1}{6}B^{-}_{\bar{1}22} & -\frac{1}{2\sqrt{6}}B^{-}_{\bar{2}11} & 0 \\
-\frac{1}{\sqrt{6}}B^{Z}_{01\bar{1}} & -\frac{1}{3\sqrt{2}}B^{Z}_{\bar{2}11} & \frac{1}{6}B^{Z}_{111} & \frac{1}{2\sqrt{3}}B^{+}_{111} & -\frac{1}{2\sqrt{6}}B^{+}_{01\bar{1}} & -\frac{1}{2\sqrt{6}}B^{+}_{\bar{2}11} & \frac{1}{3}B^{-}_{111} & 0 \\
\frac{1}{2\sqrt{2}}B^{-}_{01\bar{1}} & \frac{1}{2\sqrt{6}}B^{-}_{\bar{2}11} & \frac{1}{2\sqrt{3}}B^{-}_{111} & \frac{1}{2}B^{Z}_{111} & 0 & 0 & 0 & 0 \\
\hline
-\frac{1}{2}B^{+}_{100} & \frac{1}{2\sqrt{3}}B^{+}_{01\bar{1}} & -\frac{1}{2\sqrt{6}}B^{-}_{01\bar{1}} & 0 & -\frac{1}{2}B^{Z}_{100} & \frac{1}{2\sqrt{3}}B^{Z}_{01\bar{1}} & \frac{1}{\sqrt{6}}B^{Z}_{01\bar{1}} & \frac{1}{2\sqrt{2}}B^{-}_{01\bar{1}} \\
\frac{1}{2\sqrt{3}}B^{-}_{01\bar{1}} & \frac{1}{6}B^{+}_{\bar{1}22} & -\frac{1}{2\sqrt{6}}B^{-}_{\bar{2}11} & 0 & \frac{1}{2\sqrt{3}}B^{Z}_{01\bar{1}} & -\frac{1}{6}B^{Z}_{\bar{1}22} & \frac{1}{3\sqrt{2}}B^{Z}_{\bar{2}11} & \frac{1}{2\sqrt{6}}B^{-}_{\bar{2}11} \\
-\frac{1}{2\sqrt{6}}B^{+}_{01\bar{1}} & -\frac{1}{2\sqrt{6}}B^{+}_{\bar{2}11} & \frac{1}{3}B^{+}_{111} & 0 & \frac{1}{\sqrt{6}}B^{Z}_{01\bar{1}} & \frac{1}{3\sqrt{2}}B^{Z}_{\bar{2}11} & -\frac{1}{6}B^{Z}_{111} & \frac{1}{2\sqrt{3}}B^{-}_{111} \\
0 & 0 & 0 & 0 & \frac{1}{2\sqrt{2}}B^{+}_{01\bar{1}} & \frac{1}{2\sqrt{6}}B^{+}_{\bar{2}11} & \frac{1}{2\sqrt{3}}B^{+}_{111} & -\frac{1}{2}B^{Z}_{111}
\end{array}\right] \quad (3)$$

Here, $B^{r}_{abc}=aB^{r}_{1}+bB^{r}_{2}+cB^{r}_{3}$, where r = z, +, –, B_d denotes the magnetic field on the dth electron, and $\bar{n}$ = $-n$. The transverse magnetic field B^+ and B^- couple different S_Z subspaces with $|\Delta m_S|$ = 1, where S_Z is the spin projection to the quantization axis. Note that the spin-flip terms corresponding to $|\Delta m_S|$ = 2 are not present.

For the LZS oscillation simulation shown in Fig. 4c, 4d, we assume that 1) the transverse Overhauser field B^+ and B^- are negligibly small compared to B^Z, and 2) the spatial magnetic field gradient within a single QD is insignificant compared to that between the left and right QDs. In the (1,2) charge configuration in a DQD, we use B_L = $B_{d=1}$ to denote the magnetic field on the electron in the left QD and B_R = $B_{d=2}$ = $B_{d=3}$ to denote the magnetic field

experienced by the two electrons inside the right QD. Based on the notation and the two assumptions above, H_{hf} can be simplified as Supplementary Eq. (4).

$$H_{\mathrm{hf}} = \left[\begin{array}{cccc|cccc} \frac{1}{2}B_{\mathrm{L}}^{\mathrm{Z}} & 0 & 0 & 0 & 0 & 0 & 0 & 0 \\ 0 & \frac{1}{6}(4B_{\mathrm{R}}^{\mathrm{Z}} - B_{\mathrm{L}}^{\mathrm{Z}}) & \frac{2}{3\sqrt{2}}\Delta B_{\mathrm{Z}} & 0 & 0 & 0 & 0 & 0 \\ 0 & \frac{2}{3\sqrt{2}}\Delta B_{\mathrm{Z}} & \frac{1}{6}(B_{\mathrm{L}}^{\mathrm{Z}} + 2B_{\mathrm{R}}^{\mathrm{Z}}) & 0 & 0 & 0 & 0 & 0 \\ 0 & 0 & 0 & \frac{1}{2}(B_{\mathrm{L}}^{\mathrm{Z}} + 2B_{\mathrm{R}}^{\mathrm{Z}}) & 0 & 0 & 0 & 0 \\ \hline 0 & 0 & 0 & 0 & -\frac{1}{2}B_{\mathrm{L}}^{\mathrm{Z}} & 0 & 0 & 0 \\ 0 & 0 & 0 & 0 & 0 & -\frac{1}{6}(4B_{\mathrm{R}}^{\mathrm{Z}} - B_{\mathrm{L}}^{\mathrm{Z}}) & -\frac{2}{3\sqrt{2}}\Delta B_{\mathrm{Z}} & 0 \\ 0 & 0 & 0 & 0 & 0 & -\frac{2}{3\sqrt{2}}\Delta B_{\mathrm{Z}} & -\frac{1}{6}(B_{\mathrm{L}}^{\mathrm{Z}} + 2B_{\mathrm{R}}^{\mathrm{Z}}) & 0 \\ 0 & 0 & 0 & 0 & 0 & 0 & 0 & -\frac{1}{2}(B_{\mathrm{L}}^{\mathrm{Z}} + 2B_{\mathrm{R}}^{\mathrm{Z}}) \end{array}\right] \tag{4}$$

$$= \left[\begin{array}{c|c} H_{\mathrm{hf,P}} & 0 \\ \hline 0 & H_{\mathrm{hf,N}} \end{array}\right]$$

$H_{\mathrm{hf,P}}$ ($H_{\mathrm{hf,N}}$) is the Hamiltonian in the positive (negative) spin subspace, where $H_{\mathrm{hf,N}} = -H_{\mathrm{hf,P}}$ holds. Diagonalizing $H_{\mathrm{hf,P}}$ results in Supplementary Eq. (5) as shown below with the ordered basis [D_{S}(1,2; 1/2), D_{T0}(1,2; 1/2), D_{T1}(1,2; 1/2), Q(1,2; 3/2)] [6]. Here, D_{T0}(1,2; 1/2) = $|\uparrow\rangle|T_0\rangle$ and D_{T1}(1,2; 1/2) = $|\downarrow\rangle|T_+\rangle$, as mentioned in the main text.

$$H_{\mathrm{hf,P}} = \begin{bmatrix} \frac{1}{2}B_{\mathrm{L}}^{\mathrm{Z}} & 0 & 0 & 0 \\ 0 & \frac{1}{2}B_{\mathrm{L}}^{\mathrm{Z}} & 0 & 0 \\ 0 & 0 & \frac{1}{2}B_{\mathrm{L}}^{\mathrm{Z}} - \Delta B_{\mathrm{Z}} & 0 \\ 0 & 0 & 0 & \frac{1}{2}(B_{\mathrm{L}}^{\mathrm{Z}} + 2B_{\mathrm{R}}^{\mathrm{Z}}) \end{bmatrix} \tag{5}$$

$D_{T1} – D_{T0}$ splitting is governed by ΔB_Z, providing a direct measure of the size of the spatial magnetic field gradient, ΔB_Z. We emphasize that $D_{T0} – D_S$ splitting is now independent of the magnetic field strength, providing a decoherence-free subspace for high-fidelity qubit operations. However, we note that $D_{T0} – D_S$ splitting may still be disturbed by the magnetic field gradient noise within the right QD, which we assume to be negligible compared to ΔB_Z. After implementing the single-shot readout-based real-time Hamiltonian estimation technique[19], we anticipate that the investigation of the temporal dynamics of $D_{T0} – D_S$ splitting may enable the study of the magnetic field behavior within a single QD. This in turn would be helpful to reveal the spatial distribution of the WM wavefunction.

Supplementary Note 9. Simulation of the Landau–Zener–Stückelberg oscillation

As discussed in Supplementary Note 8, we neglect the transverse magnetic field contribution and do not consider the transition between different S_Z subspaces in the LZS oscillation simulation. This allows us to analyze the dynamics of the S_Z = 1/2 and S_Z = −1/2 subspaces separately and ignore the $|S_Z|$ = 3/2 subspace. We combine the reduced S_Z = 1/2 (S_Z = −1/2) hyperfine Hamiltonian with the electronic Hamiltonian shown in the Supplementary Note 2 to describe the dynamics in the S_Z = 1/2 (S_Z = −1/2) subspace. The hyperfine Hamiltonian in the S_Z = 1/2 subspace with the charge configurations is explicitly considered as follows. The ordered basis for the Hamiltonian is [D_S(2,1; 1/2), D_T(2,1; 1/2), Q(2,1; 1/2), D_S(1,2; 1/2), D_T(1,2; 1/2), Q(1,2; 1/2)].

$$H_{\mathrm{hf},1/2} = \left[\begin{array}{ccc|ccc} \frac{1}{2}B_{\mathrm{R}}^{\mathrm{Z}} & 0 & 0 & 0 & 0 & 0 \\ 0 & \frac{1}{6}(4B_{\mathrm{L}}^{\mathrm{Z}} - B_{\mathrm{R}}^{\mathrm{Z}}) & -\frac{2}{3\sqrt{2}}\Delta B_{\mathrm{Z}} & 0 & 0 & 0 \\ 0 & -\frac{2}{3\sqrt{2}}\Delta B_{\mathrm{Z}} & \frac{1}{6}(B_{\mathrm{R}}^{\mathrm{Z}} + 2B_{\mathrm{L}}^{\mathrm{Z}}) & 0 & 0 & 0 \\ \hline 0 & 0 & 0 & \frac{1}{2}B_{\mathrm{L}}^{\mathrm{Z}} & 0 & 0 \\ 0 & 0 & 0 & 0 & \frac{1}{6}(4B_{\mathrm{R}}^{\mathrm{Z}} - B_{\mathrm{L}}^{\mathrm{Z}}) & \frac{2}{3\sqrt{2}}\Delta B_{\mathrm{Z}} \\ 0 & 0 & 0 & 0 & \frac{2}{3\sqrt{2}}\Delta B_{\mathrm{Z}} & \frac{1}{6}(B_{\mathrm{L}}^{\mathrm{Z}} + 2B_{\mathrm{R}}^{\mathrm{Z}}) \end{array}\right] \quad \text{(SE6)}$$

For numerical reproduction of the LZS oscillation shown in Fig. 4c, we solve the time-dependent Schrödinger equation by varying the detuning parameter ε according to the pulse shape. As the state probabilistically initializes to either D_S(2,1; 1/2) or D_S(2,1; −1/2) at EST, we simulate the LZS oscillations of the S_Z = 1/2 and the S_Z = −1/2 cases separately. The simulated oscillations are then averaged assuming the equal initialization probability to D_S(2,1; 1/2) and D_S(1,2; −1/2).

Supplementary References

1. Shi, Z. *et al.* Fast coherent manipulation of three-electron states in a double quantum dot. *Nat. Commun.* **5**, 3020 (2014).
2. Kim, D. *et al.* Quantum control and process tomography of a semiconductor quantum dot hybrid qubit. *Nature* **511**, 70–74 (2014).
3. Cao, G. *et al.* Tunable Hybrid Qubit in a GaAs Double Quantum Dot. *Phys. Rev. Lett.* **116**, 086801 (2016).
4. Yannouleas, C. & Landman, U. Molecular formations and spectra due to electron correlations in three-electron hybrid double-well qubits. *Phys. Rev. B* **105**, 205302 (2022).
5. Yannouleas, C. & Landman, U. Wigner molecules and hybrid qubits. *J. Phys.: Condens. Matter* **34**, 21LT01 (2022).
6. Chen, B.-B. *et al.* Spin blockade and coherent dynamics of high-spin states in a three-electron double quantum dot. *Phys. Rev. B* **95**, 035408 (2017).
7. Hung, J.-T., Fei, J., Friesen, M. & Hu, X. Decoherence of an exchange qubit by hyperfine interaction. *Phys. Rev. B* **90**, 045308 (2014).
8. Elzerman, J. M. *et al.* Single-shot read-out of an individual electron spin in a quantum dot. *Nature* **430**, 431–435 (2004).
9. Morello, A. *et al.* Single-shot readout of an electron spin in silicon. *Nature* **467**, 687–691 (2010).
10. Jang, W. *et al.* Robust energy-selective tunneling readout of singlet-triplet qubits under large magnetic field gradient. *npj Quantum Inf.* **6**, 64 (2020).
11. Jang, W. *et al.* Single-Shot Readout of a Driven Hybrid Qubit in a GaAs Double Quantum Dot. *Nano Lett.* **21**, 4999–5005 (2021).
12. Blumoff, J. Z. *et al.* Fast and High-Fidelity State Preparation and Measurement in Triple-Quantum-Dot Spin Qubits. *PRX Quantum* **3**, 010352 (2022).

13. Petta, J. R. *et al.* Dynamic nuclear polarization with single electron spins. *Phys. Rev. Lett.* **100**, 067601 (2008).

14. Foletti, S., Bluhm, H., Mahalu, D., Umansky, V. & Yacoby, A. Universal quantum control of two-electron spin quantum bits using dynamic nuclear polarization. *Nat. Phys.* **5**, 903–908 (2009).

15. Petta, J. R. *et al.* Coherent manipulation of coupled electron spins in semiconductor quantum dots. *Science* **309**, 2180–2184 (2005).

16. Jang, W. *et al.* Individual two-axis control of three singlet-triplet qubits in a micromagnet integrated quantum dot array. *Appl. Phys. Lett.* **117**, 234001 (2020).

17. Deng, C. & Hu, X. Nuclear spin diffusion in quantum dots: Effects of inhomogeneous hyperfine interaction. *Phys. Rev. B* **72**, 165333 (2005).

18. Reilly, D. J. *et al.* Exchange Control of Nuclear Spin Diffusion in a Double Quantum Dot. *Phys. Rev. Lett.* **104**, 236802 (2010).

19. Shulman, M. D. *et al.* Suppressing qubit dephasing using real-time Hamiltonian estimation. *Nat. Commun.* **5**, 5156 (2014).
